# The *Caenorhabditis elegans* HNF4α Homolog, NHR-31, Mediates Excretory Tube Growth and Function through Coordinate Regulation of the Vacuolar ATPase

**DOI:** 10.1371/journal.pgen.1000553

**Published:** 2009-07-10

**Authors:** Annett Hahn-Windgassen, Marc R. Van Gilst

**Affiliations:** Fred Hutchinson Cancer Research Center, Seattle, Washington, United States of America; Huntsman Cancer Institute, United States of America

## Abstract

Nuclear receptors of the Hepatocyte Nuclear Factor-4 (HNF4) subtype have been linked to a host of developmental and metabolic functions in animals ranging from worms to humans; however, the full spectrum of physiological activities carried out by this nuclear receptor subfamily is far from established. We have found that the *Caenorhabditis elegans* nuclear receptor NHR-31, a homolog of mammalian HNF4 receptors, is required for controlling the growth and function of the nematode excretory cell, a multi-branched tubular cell that acts as the *C. elegans* renal system. Larval specific RNAi knockdown of *nhr-31* led to significant structural abnormalities along the length of the excretory cell canal, including numerous regions of uncontrolled growth at sites near to and distant from the cell nucleus. *nhr-31* RNAi animals were sensitive to acute challenge with ionic stress, implying that the osmoregulatory function of the excretory cell was also compromised. Gene expression profiling revealed a surprisingly specific role for *nhr-31* in the control of multiple genes that encode subunits of the vacuolar ATPase (vATPase). RNAi of these vATPase genes resulted in excretory cell defects similar to those observed in *nhr-31* RNAi animals, demonstrating that the influence of *nhr-31* on excretory cell growth is mediated, at least in part, through coordinate regulation of the vATPase. Sequence analysis revealed a stunning enrichment of HNF4α type binding sites in the promoters of both *C. elegans* and mouse vATPase genes, arguing that coordinate regulation of the vATPase by HNF4 receptors is likely to be conserved in mammals. Our study establishes a new pathway for regulation of excretory cell growth and reveals a novel role for HNF4-type nuclear receptors in the development and function of a renal system.

## Author Summary

The function of many important biological structures requires the construction of very complex cellular shapes. For example, mammalian kidneys or related renal systems in other animals rely on the formation of elongated tubes that maximize surface area to facilitate the exchange of ions between the body and excreted fluid. Defects in kidney development or function may lead to kidney failure or polycystic kidney disease. Mechanisms involved in orchestrating the formation and function of the elaborate tube structures in renal systems are still poorly characterized. Here, we show a novel transcription factor involved in the growth and elongation of an excretory tube in *C. elegans*. This factor helps manage tube development by regulating genes involved in ion transport and membrane fusion, likely helping to balance the growth of the inner and outer portions of the excretory tube as this structure elongates. This transcription factor shares significant homology with a mammalian protein that participates in hormone signaling and is present in the kidney tubules, suggesting that elongation and growth of tube structures may rely on a new kind of hormonal communication that occurs between distant parts of the cell; this signaling mechanism may be important for appropriate kidney development in humans.

## Introduction

Nuclear receptors (NRs) comprise a large family of transcription factors distinguished by a highly conserved DNA binding domain and a structurally conserved ligand-binding domain. NRs are notable for their ability to interact with small molecule ligands, enabling these factors to respond to autocrine, paracrine, and endocrine signals in order to mediate transcriptional effects at a distance [1],[2]. The canonical NR family is exclusively found in metazoans and the number of nuclear receptor members varies dramatically depending on species; from 21 NR genes in *Drosophila melanogaster*, to ∼50 in rodents and humans, to over 250 NRs in *Caenorhabditis elegans* and related nematodes [3]. The extraordinarily large NR family of *C. elegans* is particularly intriguing. Of the 283 predicted NR genes, only 15 are directly orthologous to NRs found in other metazoans, including *Drosophila* and mammals [4]. The remaining 268 NRs are thought to be derived from extensive duplication and diversification of an ancestral gene most closely related to the mammalian and *Drosophila* HNF4 receptors [5]. The presence of both highly similar and divergent HNF4-type receptors in nematodes implies that many of these proteins will carry out conserved structural and physiological functions, whereas others will have evolved to adopt responsibilities more specific to the nematode lineage. This idea is supported by the fact the *C. elegans* NHR-49 nuclear receptor shares many of the metabolic functions of the mammalian HNF4α, but not the developmental activities [6],[7]. Thus, study of *C. elegans* NRs should not only be helpful for understanding mammalian NR function and physiology, but should also reveal novel regulatory activities for the nuclear receptor family.

The prospect that the responsibilities of mammalian receptors may be divided among a larger number of NRs in *C. elegans* may be advantageous for understanding the physiological function these complex proteins. For example, the mammalian HNF4α plays numerous roles in development, metabolism, and disease [8]; because of this widespread physiological impact, the functional and mechanistic diversity of this receptor is far from understood. Indeed, mutations in the human *HNF4α* are associated with maturity onset diabetes of the young (MODY) and late onset type II diabetes; yet, how these *HNF4α* lesions lead to diabetes has not been established [9]–[11]. Furthermore, there is considerable controversy over the quantity and identity of HNF4 target genes [12]–[14]. These complications may be due, at least in part, to the fact that HNF4α carries out essential functions in several different tissues, and that HNF4α likely regulates different target sets depending on metabolic, developmental, and nutritional context.

HNF4α is also expressed in many cell types for which its function has not yet been established; for example, the epithelial cells of the intestine and the proximal and convoluted tubules of the kidney, and while HNF4α has been shown to regulate proliferation of transformed kidney cell lines, its role in kidney development remains to be defined [15],[16]. The *C. elegans* renal system is comprised of only three cells, yet these cells carry out many of the same functions as mammalian kidneys [17],[18]. Therefore, *C. elegans* might be an advantageous system in which to study the role of HNF4 receptors in renal development. The largest portion of the *C. elegans* excretory system consists of the excretory cell (EC). The development of the EC is extraordinary, as it involves the formation and growth of four branches that project outward from a single nucleus located near the anterior bulb of the pharynx [17]. These branches grow along the length of the animal to near the tip of the head and tail in early development, and then continue to grow along with the animal until adulthood. Each branch of the EC contains an inner membrane that coalesces to form a lumen; thus, the excretory cell becomes a large, single cell tube. Consequently, the EC has been effectively used to understand the development of tubes and to investigate mechanisms involved in excretory function [17],[19],[20]. At this point, factors known to participate in the development and function of the *C. elegans* excretory cell include vATPases, WNK kinases, CLIC-like proteins, Patched related proteins, and mucins [17], [21]–[24]. Additionally, the CEH-6 homeobox protein has also been implicated as the only transcriptional regulatory factor, thus far, involved in excretory cell development [25]. How the complex structure of the EC is developed and maintained so precisely, even at points very distant from the primary sites of gene regulation, remains a mystery.

We have found a highly conserved *C. elegans* HNF4 paralog, NHR-31, that is specifically expressed in the excretory cell of the nematode, suggesting that investigation of this receptor may provide unique insight into the role of nuclear receptors in renal development and tube formation. In this study, we show that NHR-31 specifically regulates the expression of genes that coordinate the synchronous growth and elongation of excretory canals, demonstrating a novel NR mediated pathway for renal system development and function.

## Results

### *nhr-31* Is Expressed in the Excretory Cell Throughout Development

**Figure 1:**
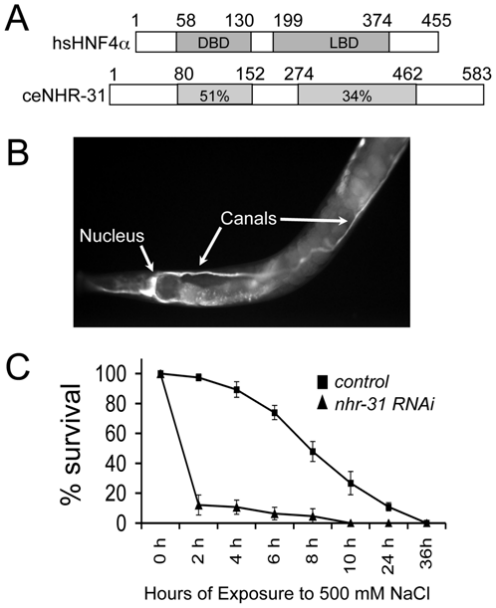
The *C. elegans* HNF4α homolog *nhr-31* is expressed in the excretory cell and is required for resistance to osmotic stress. (A) The NHR-31 protein shares significant homology with HNF4α in both the DNA (DBD) and ligand binding domains (LBD). (B) An adult animal harboring a transgenic P*_nhr-31_*::*gfp* reporter. This reporter construct showed that the *nhr-31* promoter drives strong GFP expression in the excretory cell. GFP expression in both the excretory cell nucleus and canals are indicated with arrows. There is also faint GFP expression in the intestine (C) The ability of L4 animals to survive acute exposure to 500 mM NaCl was severely compromised by reduced *nhr-31* expression. Animals fed control RNAi are shown as black squares and animals fed *nhr-31* RNAi are shown as black triangles. Data were collected using 250 animals for each time point and are presented as average number of worms surviving +/−SEM.

*nhr-31* is predicted to encode an HNF4α related nuclear receptor (NR) protein with a highly conserved DNA binding domain (DBD) and ligand binding domain (LBD) (Figure 1A). To help establish the physiological function of this NR, we determined the tissues in which the *nhr-31* gene is expressed. A GFP reporter construct was generated by fusing 3.0 kb of *nhr-31* upstream regulatory sequence to the *gfp* gene (*P_nhr-31_::gfp*). Injection of *P_nhr-31_::gfp* into WT worms revealed that the *nhr-31* promoter drives strong expression in the excretory cell (EC). In transgenic animals, GFP protein was first observed in the EC cell shortly after EC birth and persisted in the EC for the remainder of worm embryogenesis, larval development, and adulthood (Figure 1B and data not shown). GFP was observed throughout the cytoplasm of the H-shaped excretory cell. Because our reporter construct was designed by fusing only the *nhr-31* promoter to the *gfp* gene, the GFP localization pattern does not represent NHR-31 protein sub-cellular localization. *P_nhr-31_::gfp* expression was also observed, at lower levels, in the intestine and in several unidentified cells located near the tail (Figure 1B and data not shown).

### *nhr-31* Is Essential for Resistance to Osmotic Stress

In *C. elegans*, the EC functions cooperatively with duct and pore cells, and together these cells are important for maintaining osmolarity homeostasis [26],[27]. To determine if *nhr-31* RNAi animals displayed compromised excretory function, we treated animals with *nhr-31* RNAi or control RNAi from the L1 to L4 stage of development and then stressed L4 animals with acute exposure to a standard growth plate supplemented with 500 mM NaCl, and determined their ability to respond to these unfavorable conditions. 250 animals were assayed at each time point. After just two hours, less than 5% of *nhr-31* RNAi animals could be rescued from 500 mM NaCl exposure. In contrast, L4 animals fed control RNAi were able to thrive for much longer under these same conditions, with over 50% of animals maintaining the ability to recover even after 8 hours of high salt exposure (Figure 1C). These data indicate that reducing *nhr-31* gene expression strongly impairs the ability to survive acute osmotic stress.

### *nhr-31* Is Necessary for Normal Excretory Cell Development

**Figure 2:**
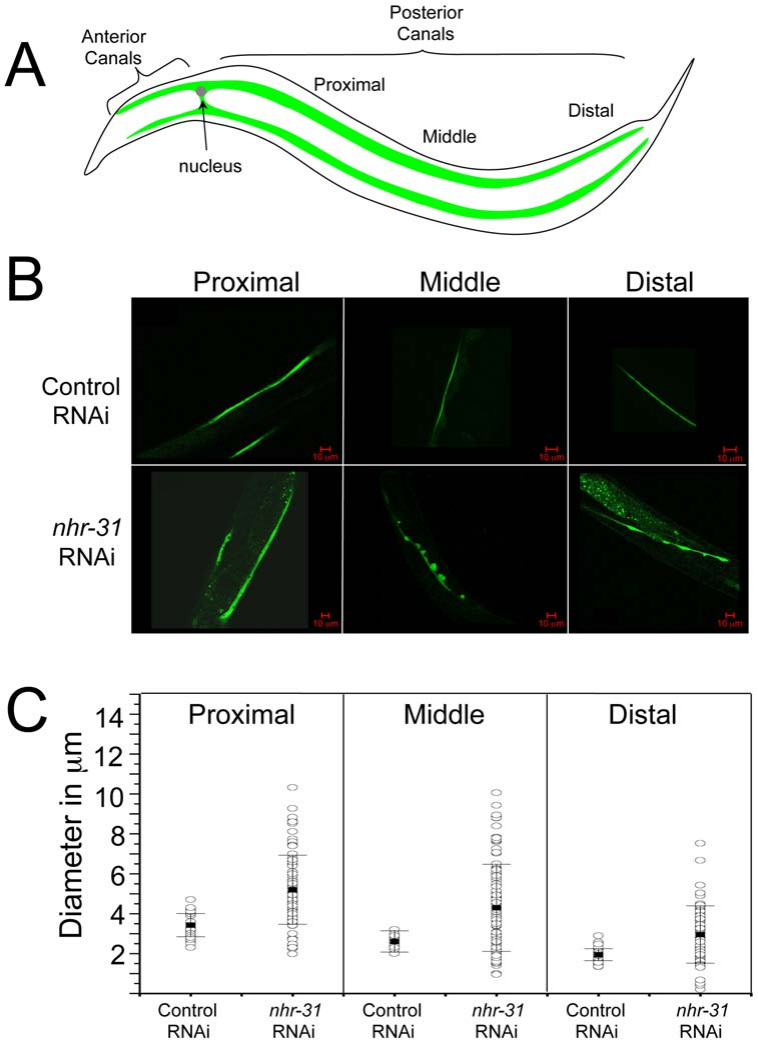
*nhr-31* is required for excretory cell morphology. (A) Diagram of the *C. elegans* excretory cell. The cell body of the excretory cell, including the nucleus, is located in the anterior part of the animal near the terminal bulb of the pharynx. Two projections extend from the cell body and each projection bifurcates into two branches. One branch runs to the posterior (tail) of the animal and the opposite branch runs to the anterior (head), building an H-shaped cell. Each projection contains an inner membrane (apical) that lines a lumen, forming a tube structure, and an outer membrane (basal), which borders and attaches to the hypodermis. Therefore, there are four tubular projections, two running down each side of the animal to the rear, and two running to the front. (B) RNAi of *nhr-31* during larval development resulted in an excretory cell that was much larger than in WT animals. *nhr-31* RNAi animals also displayed numerous enlarged areas along the entire length of the excretory cell tube; these areas are defined as “varicosities”. Excretory cell defects resulting from *nhr-31* RNAi occurred throughout the EC: shown here are the proximal, middle, and distal regions of the posterior branches. In WT adult animals, the excretory cell was uniform in diameter and did not display noticeable varicosities at any location in the EC. (C) Quantitative measurements highlight the variability in EC cell diameter in WT and *nhr-31* RNAi animals. In each control and *nhr-31* RNAi animal, the excretory cell diameter was measured in three separate 50 µm regions, one selected from the anterior portion of the worm, one from the middle, and one from the posterior region, within these regions 10 diameter measurements were obtained by imaging the EC in 5 µm intervals. 5 control RNAi and *nhr-31* RNAi animals were quantified using this strategy resulting in 50 independent diameter measurements for each EC region. To show the variation, each individual measurement is displayed here as an open circle. The black circles show the average excretory cell diameter for each measured region, and error bars represent standard deviation.

Three different *nhr-31* deletion strains have been isolated, and all of these strains are inviable (www.wormbase.org). Using one of these strains (*nhr-31(tm1547)*), we found that *nhr-31* deletion leads to early embryonic lethality (data not shown). Additionally, application of *nhr-31* RNAi throughout growth and development results in significant embryonic lethality in the F_1_ generation (data not shown). Thus, NHR-31, like its mammalian homolog HNF4α, plays an essential role in early embryonic development. Because we found that the *nhr-31* gene is primarily expressed in the excretory cell during larval and adult stages, however, we investigated the participation of *nhr-31* in EC development and morphology using an RNAi feeding strategy that specifically reduced *nhr-31* expression during larval development and adulthood. In postembryonic animals, the EC is an H-shaped cell, with four canals emanating from a main cell body located near the terminal bulb of the pharynx [17]. Two canals project along each side of the animal towards the posterior end, and two canals project forward towards the anterior end (Figure 2A). To monitor EC morphology, WT animals were injected with the *P_nhr-31_::gfp* reporter. In WT adult animals, GFP localization revealed that the outer diameter of the excretory cell was relatively uniform through the entire length of the canal, measuring ∼3.5 µm in proximal sections of the posterior canal, and tapering to ∼2.4 µm in distal sections of the posterior canal (Figure 2B and 2C).

When WT animals carrying the *P_nhr-31_::gfp* construct were treated with *nhr-31* RNAi from the L1 stage of larval development through adulthood, the morphology of the adult EC was dramatically altered (Figure 2B and 2C). In particular, the excretory canals were not uniform in diameter; instead, they contained multiple enlarged varicosities, with diameters up to 10 µm (Figure 2B and 2C). These varicosities showed considerable variability in size and shape and were located along the entire length of the EC, including the proximal, middle, and distal portions of the posterior arms, as well as in the anterior branches of the EC canal (Figure 2B and 2C and data not shown). DIC images of *nhr-31(+/−)* heterozygotes also revealed similar excretory cell abnormalities, providing support for the specificity of our *nhr-31* RNAi construct (Figure S1).

**Figure 3:**
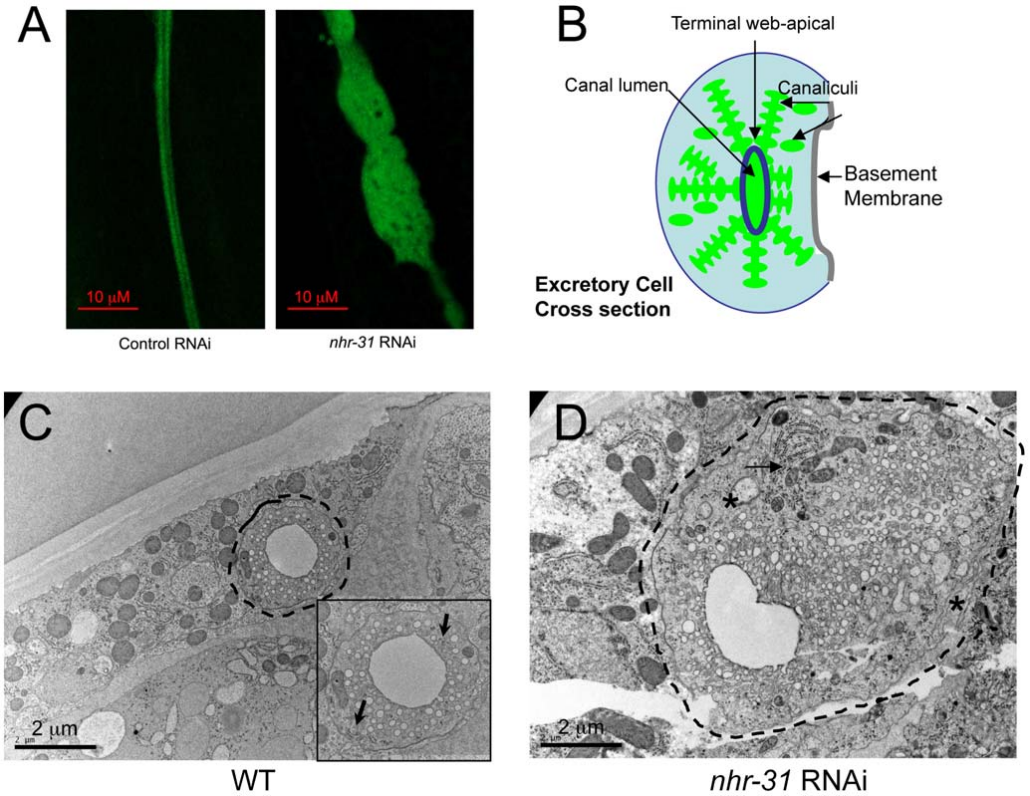
Knockout of *nhr-31* results in uncontrolled cellular growth. (A) High magnification confocal images revealed that the varicosities in the *nhr-31* RNAi animals appeared to be dense with cytoplasm and punctuated with vacuole like structures. (B) Illustration of an excretory cell cross-section. Excretory cell tubes harbor an apical membrane that forms the lumen; the surface area of the inner lumen is significantly enhanced by numerous canaliculi that branch off of the center lumen. (C) High pressure freezing transmission electron microscopy (HP-TEM) images of animals subjected to control RNAi. The excretory cell resides within the black dotted line, which is drawn just outside of the EC membrane. For better visibility, the EC is also shown enlarged in an inset. Control RNAi animals displayed a well-formed lumen with small regions of endoplasmic reticulum (ER) on both sides of the EC (the ER is indicated with black arrows in the inset). The outer diameter of the excretory cell was approximately 2.0–2.5 µm (depending on the axis), and the diameter of the lumen was approximately 1.6 µm normal diameter. Furthermore, the lumen was surrounded by well-formed canaliculi (illustrated in black rectangle), which appear as circular structures around the lumen. (D) A TEM image of an *nhr-31* RNAi animal at the same magnification. This section was taken through a large abnormal varicosity. This excretory cell harbors more abundant ER (black arrows), mitochondria and canaliculi, as well as large intracellular vesicles (indicated with an asterisk). As clearly shown in this TEM image, *nhr-31* RNAi animals also displayed poorly defined basal membranes (visible just inside the dotted line). In this image, the cell and lumen diameters were significantly greater than those of a WT excretory cell. Larger images are available in the online supplementary data section (Figure S2).

High magnification of the GFP images obtained in *nhr-31* RNAi animals suggested that the varicosities consisted of dense cellular material with an abundance of vacuoles (Figure 3A). This phenotype was different from previously reported EC abnormalities, which showed enlargement of the EC cell due to fluid accumulation or cyst formation [19],[27]. To more closely examine the morphological defects in the EC of *nhr-31* RNAi animals, we employed high pressure freezing transmission electron microscopy (HP-TEM). Table 1 shows quantitative analysis of sections obtained from the middle region of the EC in 5 different control RNAi animals and 5 different *nhr-31* RNAi animals. Cross sections of the EC of a WT animal showed a single circular lumen with an average diameter of 1.6 µm (Table 1). Additionally, an abundance of well-formed canaliculi were clearly visible in WT animals (Figure 3C and Table 1). Canaliculi are smaller “mini-canals” surrounding the canal lumen; these canals are thought to greatly increase the apical surface area of the EC lumen (Figure 3B) [17]. Canaliculi were visible in the wild type excretory canal cross section as small, round, circular shapes and were regular in size and consistent (∼70/section) in number from section to section (Figure 3D and Table 1). According to our EM measurements, the average diameter of the EC was ∼2.8 µm, which agreed nicely with our GFP measurements (Figure 2C and Table 1).

**Table 1:** Analysis and Quantification of TEM Excretory Cell Images.

Section #	Cell Diameter (mm)	Lumen Diameter (mm)	# Ectopic Vesicles	Total # Mitochondria	ER Abundance	Canaliculi
**Control RNAi**						
1	3	1.6	0	2	+	57
2	2.5	1.5	0	1	+	63
3	2.8	1.5	0	1	+	64
4	3.5	2	1	4	+	93
5	2	2	0	2	+	81
6	2.5	1.5	0	2	+	64
7	3	1.5	0	2	+	78
8	2.5	1.3	0	4	+	53
9	3	1.3	0	1	+	71
10	2.7	1.3	0	2	+	64
Average	**2.8**	**1.6**	**0.1**	**2.1**		**68.8**
***nhr-31*** ** RNAi**						
1	8	2	6	22	+++	115
2	8	1.5	10	20	+++	116
3	8	2.5	13	12	+++	156
4	8	4	26	27	+++	408
5	8	1.5	0	7	++	74
6	8	4.2	3	13	+++	118
7	6	2	3	4	+++	106
8	4	1.4	1	6	++	108
9	4	1.2	0	6	+++	102
10	3	0.7	1	3	−	26
11	2	1.5	2	4	−	101
12	2	0.5	0	2	−	77
Average	**5.8**	**1.9**	**5.4**	**10.5**		**125.6**

High-pressure TEM sections were prepared from young adult animals exposed to control RNAi and *nhr-31* RNAi. Images are sorted from sections showing the highest EC diameter to the lowest. For ER abundance, (+) is equal to normal ER abundance, whereas (++) and (+++) represent above normal and much above normal, respectively. (−) indicates below normal ER abundance.

HP-TEM imaging revealed multiple morphological defects in the excretory canals of *nhr-31* RNAi animals, particularly in the varicosities (Figure 3D and Table 1). First, the average canal diameter increased to 5.8 µm, with larger varicosities displaying diameters of up to 8 µm, and the narrow regions showing diameters from 2–3 µm. Second, the average diameter of the lumen in *nhr-31* RNAi animals was increased by 26% to 1.95 µm, and the lumen often appeared multi-lobed. The diameter of the lumen correlated strongly with the outer cell diameter, as the largest lumen diameter measurements were found within large varicosities (Table 1). Third, we found that the canaliculi were uncharacteristically irregular in size and present at much higher numbers (∼126/cell) in *nhr-31* RNAi animals (Figure 3D and Table 1). Finally, the varicosities of *nhr-31* RNAi animals possessed an unusually high number of large vesicles, elevated endoplasmic reticulum abundance, and a considerable increase in mitochondria (Figure 3E and Table 1). Importantly, the TEM cross sections showed that the varicosities were not a result of an EC canal lumen that was folded back on itself or bent away from the normal lateral alignment, or due to osmotic “swelling”, both of which have been previously reported for mutants that affected EC structure [19],[27]. Consequently, the EC phenotypes resulting from loss of *nhr-31* function are different from previous observations and suggest that *nhr-31* defects are distinctive in their mechanism of origin. In summary, both fluorescence confocal microscopy and TEM showed that loss of *nhr-31* function leads to significant defects in EC canal size, shape, and microstructure. The abundance of cellular material and organelles, along with significant structural abnormalities, implies that the abnormal varicosities observed in adult *nhr-31* RNAi animals are likely to result from regions of uncontrolled cellular growth.

### NHR-31 Coordinately Regulates Expression of Vacuolar ATPase Genes

We next applied gene expression profiling to establish downstream regulatory targets of *nhr-31*. Gene expression was measured using *C. elegans* oligomer based microarrays. We carried out this study in L4 larvae, as this is the larval stage at which the EC morphology differences between WT animals and *nhr-31* RNAi animals first begin to show. Overall, we found that, in *nhr-31* RNAi worms, the expression of 20 genes was suppressed by greater than 2-fold and the expression of 63 genes were enhanced by greater than 2-fold (Table S1).

**Figure 4:**
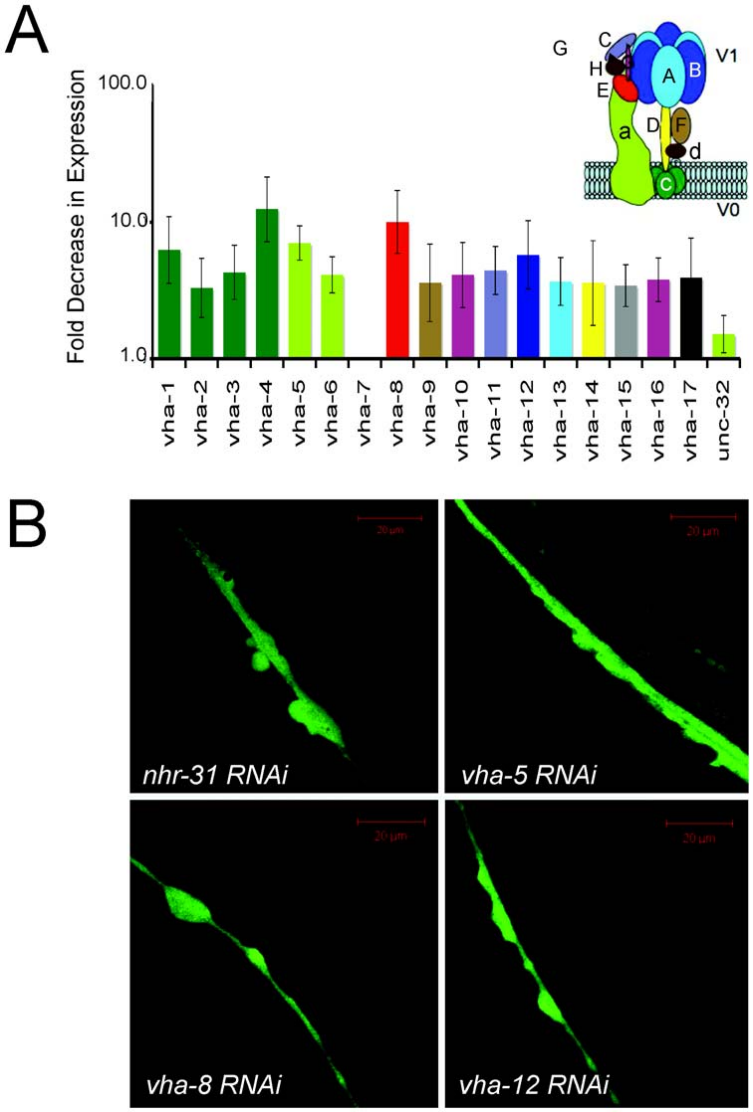
NHR-31 promotes expression of numerous genes encoding subunits of the vacuolar ATPase. (A) Change in the expression of vacuolar ATPase genes (*vha* genes) in *nhr-31* RNAi animals. Data were obtained by QRT-PCR and are plotted as the fold-decrease in gene expression observed in *nhr-31* RNAi animals as compared to animals fed control RNAi. The data are presented on a log scale and are color-coded according to the specific subunit encoded by that gene (the vATPase holoenzyme is shown in the inset, the peripheral domain is named as the V_1_ domain and the integral membrane domain is termed V_0_). For some subunits, several different gene isoforms exist. Error bars represent standard error (n = 7). (B) RNAi of vacuolar ATPase subunits revealed tube formation phenotypes similar to those of *nhr-31* RNAi animals. To avoid the larval lethality that occurs when *vha* subunits are disrupted very early in development, we treated worms with *nhr-31*, *vha-5*, *vha-8 and vha-12* RNAi beginning at the L3 stage of larval development. In each confocal image, the middle region of a posterior excretory cell canal is shown. RNAi of all four genes yielded excretory cells punctuated by abnormally shaped and large varicosities.

The most striking outcome of our microarray experiments was the discovery that RNAi of *nhr-31* dramatically affected the expression of 15 genes that encode subunits of the vacuolar ATPase (gene names are referred to as *vha*), and one gene predicted to code for a vATPase cofactor (gene name, R03E1.2). In fact, of the 30 genes most strongly reduced by inhibition of *nhr-31*, 15 of these were *vha* genes (Table S2). The vacuolar ATPase (vATPase) is an ATP-dependent proton pump, which transports protons across cellular membranes (Figure 4A). Each *C. elegans vha* gene encodes for one subunit of the holoenzyme, and there are 15 separate subunits that make up the holoenzyme. For several of the vATPase subunits, *C. elegans* possesses multiple gene isoforms; consequently there are 18 *vha* genes in total. As a secondary confirmation of the microarray data, we employed quantitative RT-PCR to specifically measure the mRNA levels of all 18 vATPase genes found in *C. elegans*. We found that the expression of 16 of these genes was reduced when *nhr-31* was inhibited (Figure 4A). Importantly, previously published data show that nearly all *vha* subunits are expressed in the excretory cell, indicating NHR-31 is likely to be mediating expression of these *vha* genes directly in the EC (Table 2) [19], [20], [29]–[33]. Additionally, most *vha* genes are also expressed in the intestine, where NHR-31 also resides. Accordingly, the only two *vha* genes not regulated by NHR-31, *vha-7* and *unc-32*, are not expressed in the excretory cell. In sum, our microarray and QRT-PCR convincingly demonstrate that a primary function of NHR-31 is to coordinately promote the expression of almost the entire complement of vacuolar ATPase genes. NHR-31 localization to the excretory cell, where nearly all *vha* genes are expressed, also argues that NHR-31 is regulating *vha* genes in this cell type.

**Table 2:** Expression Patterns of *C. elegans vha* Genes.

*vha-1*	expressed in **excretory cell** in larvae and adults
*vha-2*	expressed in **excretory cell** in larvae and adults
*vha-3*	intestine, hypodermis, and **excretory cel**l
*vha-4*	**excretory cell**
*vha-5*	**broadly in embryo, excretory cell**, pharynx, and some hypodermal cells
*vha-6*	intestinal cells
***vha-7***	mature gonad, spermatheca
*vha-8*	**excretory cell**
*vha-9*	N/A
*vha-10*	N/A
*vha-11*	**excretory cell** and intestine
*vha-12*	pharynx, intestine, and **excretory cell**
*vha-13*	intestine, body wall muscle, and **excretory cell**
*vha-14*	N/A
*vha-15*	widely expressed, including **excretory cell** and intestine
*vha-16*	widely expressed, including **excretory cell** and intestine
*vha-17*	**excretory cell**, intestine, and epidermal cells
***unc-32***	gonad, intestine, and many neuronal cells

Expression patterns of the vha genes were obtained from previously published studies [19], [20], [29]–[32]. *vha-6* and *unc-32* are indicated in bold because they are not regulated by *nhr-31*.

### Vacuolar ATPase Subunits Are Required Late in Larval Development

Because the vacuolar ATPase subunits are highly expressed in the EC, we suspected that the impact of *nhr-31* on EC development might be a consequence of vacuolar ATPase regulation. To test this hypothesis, we used RNAi feeding to specifically reduce the expression of three different vacuolar ATPase subunits: *vha-5* (small *a* subunit), *vha-8* (catalytic E subunit) and *vha-12* (B subunit). Because previous studies have shown that RNAi of the vacuolar ATPase subunits leads to larval lethality, we did not apply *vha* or *nhr-31* RNAi until the L3 stage of development. Using this approach, we found that RNAi of each of these subunits was sufficient to cause excretory canal formation defects similar to those of *nhr-31* RNAi animals (Figure 4B). These results imply that the control of EC development by NHR-31 is mediated, at least in part, by its stimulation of vATPase expression. We also note that this experiment shows that knockdown of *nhr-31* or vATPase expression specifically in late larval development is sufficient to cause irregular EC growth and adult varicosities.

### Abnormal Varicosities in *nhr-31* RNAi Worms Arise Late in Development

**Figure 5:**
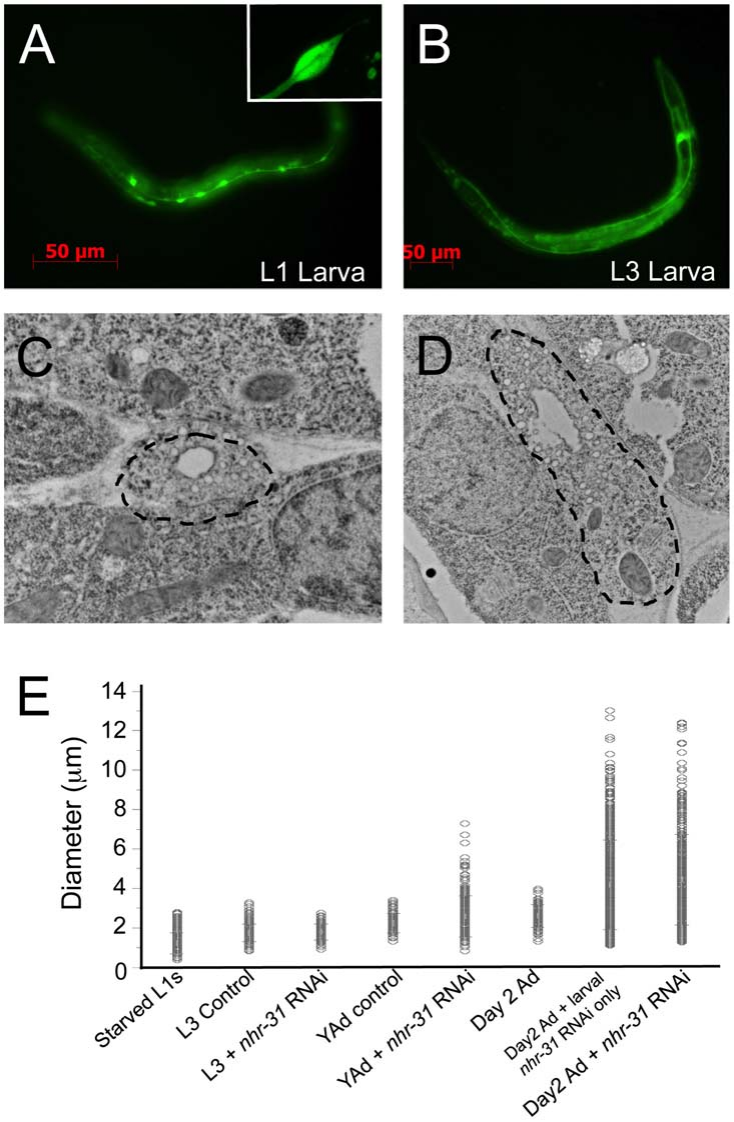
In WT animals, varicosities are present during excretory cell development and disappear in adult animals. (A) Although large and irregular varicosities were not observed in WT adult animals, we found that varicosity structures were common in early larval development. In L1 larvae, varicosities were present at regular intervals along the entire length of the excretory cell. (B) By the L3 stage of development, these developmental varicosities decreased in size such that they were no longer easily visible. (C,D) A TEM image of a narrow region (C) and a varicosity (D) in the EC of a WT animal during the L1 stage of larval development. Like the varicosities observed in *nhr-31* RNAi adults, the L1 varicosities contained extra cellular material, with more canaliculi and larger mitochondria (see also Table 3). Larger EM images are available in the supplement (Figure S3). (E) Quantification of EC diameters when exposed to RNAi at different points in development. Graph includes diameters measured using the P*_nhr-31_::gfp* strain and confocal microscopy. The graph contains measurements of the EC of animals exposed to the following treatments: Measurement of L1 larvae starved for 12 hours (starved-L1), measurement of L3 ECs when exposed to control RNAi (L3-control) or *nhr-31* RNAi (L3+*nhr-31* RNAi) from the L1 to the L3 stage of development. Measurement of L4/young adult ECs when worms are exposed to control (L4/YAd control) or *nhr-31* RNAi (L4/YAd+*nhr-31* RNAi) from the L1 to the L4 stage. Animals exposed to control RNAi (Day 2Ad Control) or NHR-31 RNAi (Day2 Ad+larval *nhr-31* only) from L1 to 2 day old adults, and then switched to control RNAi, and animals exposed to *nhr-31* RNAi from L1 until day 2 of adulthood (day 2Ad+nhr-31 RNAi). This data shows that exposure to *nhr-31* RNAi from the L1 stage of development to the L3 stage of development does not impact EC size in L3 worms, but that varicosities appear in late L4 development and continue to grow into adulthood.

Although the large, irregular, varicosities observed in *nhr-31* RNAi animals were never observed in WT adults, we did notice varicosity-like structures early in WT larval development, residing at regular intervals along the EC canal in L1 and early L2 animals (Figure 5A and Table 3). These varicosities differed from those present in *nhr-31* RNAi adults in that they displayed a consistently symmetrical oval shape (Figure 5A). In L1 larvae, ∼10 of these varicosities were observed in each EC canal branch, but as worms developed the regions of the excretory cell between varicosities grew wider and the varicosities consequently decreased in prominence such that, by the late L3 stage of development, the entire length of the excretory cell possessed a diameter similar to the varicosities observed in L1 animals (Figure 5B and 5E). The presence of these growth varicosities in WT L1 larvae was confirmed by hp-TEM (Figure 5C and 5D). According to these TEM measurements, L1 varicosities displayed a diameter that was 2.8 times that of narrow regions, and a lumen diameter that was about 2-fold larger than the narrow regions (Table 3). Additionally, the varicosity regions harbored many more canaliculi (Figure 5C and 5D and Table 3). This data implies that the varicosities may form in L1 animals and spread horizontally along the excretory cell to help increase cellular diameter, and perhaps also length. Thus, we suspected that *nhr-31* RNAi animals might improperly maintain these structures such that they continue to enlarged and become irregularly shaped as animals developed into adults. However, examination of *nhr-31* RNAi animals revealed no obvious signs of varicosities in the L3 stage of development, implying that knockdown of *nhr-31* did not interfere with the normal dissipation of these structures during mid-larval development (Figure 5E). The varicosities that arise in *nhr-31* RNAi animals first appear in the late L4 stage of development and continue to grow larger as animals grow older (Figure 5E). Consequently, the varicosities observed in adult *nhr-31* RNAi animals must either occur from growth of new structures, or the reactivation and renewed growth of these original varicosities. We also note that the varicosities caused by *nhr-31* loss of function continue to grow larger during adulthood, such that by day 2 of adulthood they are nearly twice as large as varicosities in early adults (Figure 5E).

**Table 3:** Analysis of TEM Excretory Cell Images From WT L1 Larvae.

Section #	Cell Diameter (mm)	Lumen Diameter (mm)	Total # Mitochondria	Canaliculi
**L1 Narrow Regions**				
1	1.21	0.34	1	9
4	1.51	0.37	1	14
6	1.34	0.37	1	14
8	1.57	0.36	1	15
10	1.55	0.34	1	10
12	1.73	0.37	2	12
Average	**1.49**	**0.36**	**1.17**	**12.3**
**L1 Varicosities**				
3	4.4	0.6	1	28
5	4.2	0.9	2	35
9	3.4	0.6	1	23
11	4.3	0.7	1	27
Average	**4.1**	**0.7**	**1.3**	**28.3**

High-pressure TEM sections were prepared from WT L1 larvae. Sections were taken through narrow regions of the EC and through varicosities.

### NRE Response Element Prediction in Nematode and Mammalian vATPase Genes

**Figure 6:**
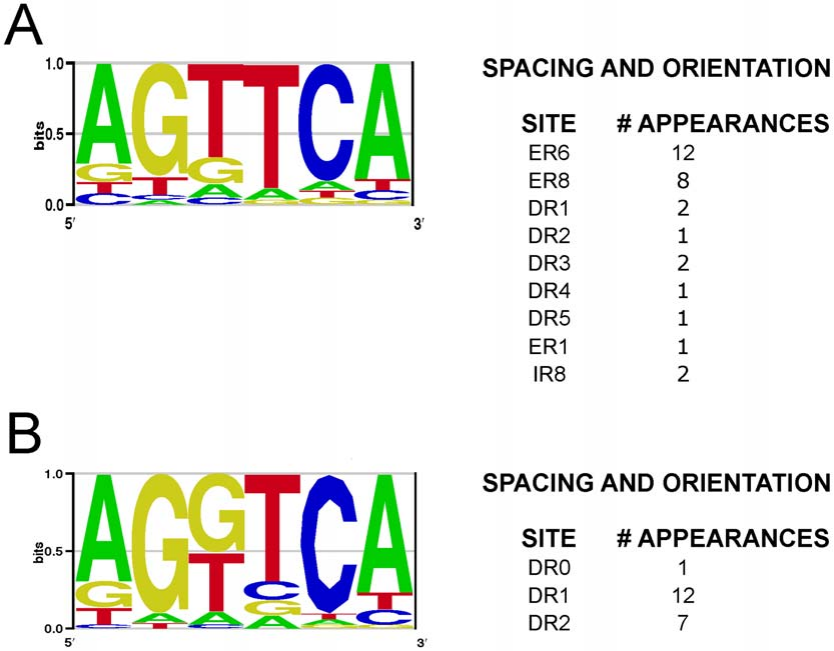
Identification of Nuclear Receptor Response Elements in *C. elegans* and mouse. Half-sites were taken from the NREs identified using NHR-scan and displayed in Table 3. These sites were analyzed using WebLogo software [43], and the consensus half-site determined by that program is displayed here for (A) *C. elegans* vATPase genes and (B) for mouse vATPase genes. The frequency of each type of response element is also shown in this figure.

Nuclear receptors typically associate with complex binding motifs comprised of two hexameric half-sites [2]. These half sites may be paired in multiple orientations with various amounts of spacing, and this architecture helps determine the type of NHR that binds. To identify NREs in the promoters of the *C. elegans* vacuolar ATPase genes, we used the NHR-computational analysis program “NHR-scan” [34]. This program identified strong NRE candidates in nearly all of the *vha* promoters; 15 out of 18 *vha* genes harbored candidate NREs in close proximity to their transcription start site. If a *vha* gene was expressed as part of an operon, NREs were found near the transcription start site of the first gene in the operon. Analysis of predicted NREs showed a strong presence of an AGTTCA consensus half site (Figure 6A and Table 4). The most common repeats were an ER6 (40% of all binding sites), which is an everted repeat separated by 6 base pairs and an ER8 (27% of all binding sites), an everted repeat separated by 8 base pairs. In fact, 13 of 19 vATPase genes had at least one highly conserved ER6 or ER8 site in their promoters, while several other types of AGTTCA repeats were also found once or twice in vATPase promoters. Interestingly, we also found a consensus ER6 site in the *nhr-31* promoter, implying that *nhr-31* may regulate its own expression through a feedback or feed-forward mechanism. This putative regulation did not manifest in our GFP reporter studies, however, implying that self-regulation in the excretory cell is not very significant during development.

**Table 4:** Predicted NRE Sites in Nematode and Mammalian vATPase Promoters.

Predicted NRE Sites in *C. elegans* Vacuolar ATPase Genes					
Promoter	Repeat Type	Sequence	Match	Orientation	Location
*vha-1/vha-2*	DR2	**AGTTCA** ta **AGTaCA**	11/12	Reverse	−612
	ER8	**TGACCg** tttttatt **AGTTCA**	11/12	Reverse	−66
*vha-3/vha-11*	ER8	**TGtCCT** tcgcataa **AGaTCA**	10/12	Forward	−2612
*vha-4*	ER1	**TtACCT** t **cGTTCA**	10/12	Forward	−528
	ER8	**TtACCT** ttcagaga **AGTTgA**	10/12	Forward	−625
*vha-5*	ER8	**TGAAag** tttggatt **AGTTCA**	10/12	Forward	−729
	ER6	**aGAACT** gtgaga **AtTTCA**	10/12	Forward	−286
*vha-6*	ER6	**TGAACT** tgtaag **gcTTCA**	10/12	Forward	−306
	ER6	**TGTAgT** caaaag **AGcTCA**	10/12	Forward	−729
*vha-7*	DR1	**AGGtAA** a **cGTTCA**	10/12	Reverse	−2283
	ER6	**TGAAaT** tcaact **AtTTCA**	10/12	Forward	−1036
*vha-9*	IR8	**AGcTtt** acagattc **TGAgCT**	8/12	Forward	−537
	ER6	**gGTTCA** attttc **TGAtaT**	9/12	Forward	−2102
*vha-10*	ER8	**TGAAaT** tctaaaat **AtTTCA**	10/12	Forward	−663
	ER8	**TGACtT** ttagttaa **aGTTCa**	9/12	Forward	−319
*vha-12*	DR1	**gGTTCA** t **cGGTCt**	9/12	Forward	−219
	DR4	**gGGTCt** tcat **gGTTCA**	9/12	Reverse	−583
	DR5	**AGTTCA** aaaat **tGTTCA**	11/12	Forward	−1928
	ER8	**TGaACc** taaaaatc **AGTTCA**	10/12	Forward	−2448
*vha-13*	ER8	**TGAACT** ccgttcga **AaTTCc**	10/12	Forward	−458
	ER6	**TcAACT** aatttt **tGTTCA**	10/12	Forward	+241
	ER6	**TGAACa** aaaatt **AGTTgA**	10/12	Forward	+1028
*vha-14*	DR3	**AGTTCg** tgg **AGTTCA**	11/12	Forward	−3068
*vha-16*	ER6	**TGgaCT** ttcgga **AGTTaA**	9/12	Forward	−307
*vha-17*	ER6	**TGAACT** gatgga **AtgTCg**	9/12	Forward	−1570
*nhr-31*	ER6	**TGAtCT** acgaat **AGTTCA**	11/12	Forward	−4788

Predicted HNF4α Sites in Mouse Vacuolar ATPase Genes					
Promoter	Repeat Type	Sequence	Match	Orientation	Location
*Atp6voe-008*	DR1	**AGTTCA** t **gGTgCA**	10/12	Forward	−999
	DR2	**AGGTCc** aa **AGTTCA**	11/12	Reverse	−1361
*Atp6v0c*	DR2	**AGaTCA** tg **AGTTCA**	11/12	Forward	−798
*Atp6v0d1*	DR1	**AGGTCt** c **tGGcCA**	9/12	Reverse	−726
	DR1	**tGGaCt** c **AGGTCA**	9/12	Reverse	−1028
*Atp6v0a2*	DR1	**AGGaCA** t **tGGTCc**	9/12	Reverse	−1573
	DR2	**ctTTCA** ta **AGTTCA**	10/12	Reverse	−775
*Atp6v1a-007*	DR1	**gGGTgA** t **AGGTCA**	10/12	Reverse	−1182
	DR1	**gGGTtA** a **AGTgCA**	10/12	Reverse	−1741
*Atp6v1h-007*	DR1	**AGGTCA** t **AaacCA**	9/12	Forward	−1748
	DR1	**tGTaCt** a **AGGTCA**	9/12	Forward	−243
*Atp6v1c2*	DR2	**gGaTCA** tt **AGTTCA**	10/12	Reverse	−772
	DR2	**AGGTCc** tg **AGTTCA**	11/12	Forward	−1232
*Atp6v1c1*	DR0	**AGTTCA AGGTCA**	12/12	Forward	−1327
	DR1	**gGcTCA** c **AGTTCA**	10/12	Forward	−517
	DR2	**gGTTCA** ac **AGGTaA**	10/12	Reverse	−797
	DR1	**AGTgCA** g **AaGTCA**	10/12	Forward	−1719
*Atp6v1b2*	DR2	**AGGTCc** ag **AGTTCA**	11/12	Reverse	−587
	DR1	**gGGcCA** c **AGGgCA**	10/12	Reverse	−1732
*Atp6v1g2*	DR1	**AGGcCg** g **AGGcCt**	8/12	Forward	−1152

Response elements were identified using the NHR-scan computational program (nhrscan.genereg.ne). Predicted NHR-31 and HNF4 response elements are shown along with their correlating vATPase genes. Nucleotides matching the consensus sequence are shown in capital letters. Location refers to the distance of the response element from the predicted ATG translational start site.

The most common spacing for mammalian HNF4α receptors is a DR1 or DR2, however it would not be surprising if NHR-31 adopted a different NRE specificity, as nematode NR binding sites have likely evolved to generate NREs to help distinguish between all of the different HNF4 paralogs in *C. elegans*. Consistent with this notion, the NHR-31 LBD does not retain two conserved amino acids that help direct HNF4α homodimerization on DR1 and DR2 sites [35]. It is also possible, however, that everted repeats have not yet been widely characterized as HNF4α sites in other organisms. The presence of so many binding sites that closely match a consensus site is quit remarkable, especially since NHR response elements are notoriously degenerate [36]. Furthermore, nuclear receptor regulated genes often contain several conserved and cryptic NREs that are necessary for modulating expression level, consequently, there are likely to be important cryptic NREs in these promoters as well [37].

Analysis of the vATPase gene promoters from mice (*Mus musculus*) showed an astonishing enrichment of HNF4α binding sites (Table 4). In fact, we found highly conserved HNF4α binding sites in 10 vacuolar ATPase genes, and most of these genes harbored at least two independent HNF4α binding sites. The repeats were almost always in DR1 or DR2 configuration and the consensus half-site sequence for these sites was AG(G/T)TCA (Figure 6B), which matches the consensus site previously reported for HNF4α binding sites [38]. As with the *C. elegans* NREs, the enrichment of these binding sites is highly significant.

Taken together, these data strongly argue that coordinate regulation of vacuolar ATPase genes by the HNF4 nuclear receptor is conserved in mammals. We should note, however, that the DR1 and DR2 elements can also bind other mammalian nuclear receptors; therefore, even though NHR-31 is most closely related to HNF4α, and expressed along with vATPases in the excretory system, the participation of other mammalian nuclear receptors in coordinate regulation of vATPase genes cannot be ruled out. Similarly, we cannot rule out the involvement of additional *C. elegans* NRs in regulation of nematode vATPase genes.

## Discussion

### NHR-31 Control of the Vacuolar ATPase Is Critical for Excretory Cell Development

We have identified a new pathway involved in the development of the *C. elegans* renal system. In summary, we have shown that the NHR-31 nuclear receptor, through promotion of vacuolar ATPase gene expression, is essential for the appropriate growth, morphology, and function of the *C. elegans* excretory cell. This study not only identifies a new transcriptional regulator necessary for EC development, but also establishes the specific regulatory targets that mediate its effects, and highlights potential nuclear receptor response elements. The regulatory or developmental activities carried out by NHR-31 have not yet been observed for a nuclear receptor; consequently our findings expand the physiological repertoire of the NR superfamily.

A primary function of NHR-31 is to maintain the structure of the EC canal during the transition from larval development into adulthood. When exposed to *nhr-31* RNAi throughout larval development, or specifically in late larval development, we observed numerous large and irregular varicosities all along the length of the posterior and anterior EC canals, these varicosities first manifested in L4 development and continued to amplify and grow several days into adulthood. As the excretory cell is involved in the regulation of ion transport and osmolarity, we considered that these varicosities might have been due to accumulation of fluid within the EC cytoplasm to create “cyst-like” structures. However, HP-TEM revealed numerous sub-cellular abnormalities within the varicosities that could not be explained by an abnormal accumulation of fluid. For example, *nhr-31* RNAi dependent varicosities generally contained abnormally shaped lumens, significant increases in the number of canaliculi, ER and mitochondria, and abnormally large numbers of ectopic vesicles. These data imply that the EC varicosities are not fluid filled, but rather overdeveloped. In contrast, in the narrow regions of the *nhr-31* RNAi EC, we found normal numbers of mitochondria, ER, and canaliculi, implying the majority of EC irregularities that occur in *nhr-31* RNAi animals are localized to the enlarged varicosities. This excessive growth phenotype significantly differs from previously characterized excretory cell phenotypes [19],[27].

Another intriguing finding of our study is that NHR-31 has a surprisingly specific and strong impact on the expression of v-ATPase encoding genes (*vha* genes). The vacuolar ATPase (v-ATPase) is an ATP-dependent proton pump that is organized into a peripheral domain (V_1_), which is responsible for ATP hydrolysis, and an integral domain (V_0_), responsible for proton transport. Although it is referred to as the vacuolar ATPase, this enzyme is found in multiple intracellular membranes, including endosomes, lysosomes, Golgi-derived vesicles, clathrin coated vesicles, secretory vesicles, as well as the plasma membrane [39],[40]. vATPases are important for numerous cellular functions, including ion transport, substrate transport, acidification of vesicles and other organelles. Additionally, recent studies have shown that vATPases also play a predominant role in vesicular trafficking of the endocytic and exocytic pathways, participating directly in membrane fusion by not only providing the proper acidic environment, but also by directly forming protein complexes during the fusion process [40]. Given the diversity of vATPase functions, it seems likely that the transcription of vATPase would be precisely regulated both spatially and temporally in order to facilitate the development and function of different cell types. Although numerous factors have been shown to regulate the vATPase at the enzymatic level, our study has identified a transcription factor with a specific role in regulating vATPase expression in a tissue specific manner.

In *C. elegans* it has been shown that vATPase subunits of either the V_0_ sector or the V_1_ sector, are important in excretory cell development and morphology [19]. In this previous study, several distinct *vha* subunits were knocked down early in development resulting in several defects in the hypodermis, cuticle, and excretory cell. Specifically, abnormal structures were observed in the ECs that were described as “whorls”. Because RNAi of *nhr-31* leads to the reduced expression of 17 out of the 19 genes that encode *vha* subunits, we suspected that the role of *nhr-31* in EC development may be due, at least in part, to regulation of vATPase gene expression. In support of this hypothesis, we found that larval specific knockdown of NHR-31 target genes encoding either an *a* subunit, an E subunit, or a B subunit of the vATPase, led to excretory cell phenotypes nearly identical to those observed in *nhr-31* RNAi animals. Although varicosities found in our experiments may be related, in some fashion, to the “whorls” observed in the previous study ([19], it should be noted that the previous study focused on reduction of vATPase expression much earlier in development. In contrast, in our study, *vha* expression was knocked down specifically in late L3 development through early adulthood. Thus, our findings show that regulation of vATPase expression is a prominent factor in NHR-31 function.

### Model for the Role of NHR-31 in Excretory Cell Development

**Figure 7:**
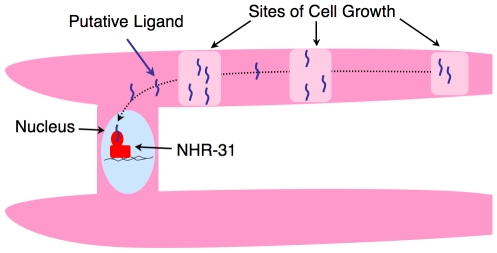
Model for NHR-31 control of EC growth and morphology. We propose that EC growth and elongation is mediated by regions of high growth activity, visible as varicosities in young larvae, but which normally dissipate as the EC reaches its full length. In *nhr-31* RNAi animals, regulation of these growth regions is disrupted, leading to uncontrolled growth and thereby formation of ectopic varicosities.

The phenotypic abnormalities observed in *nhr-31* RNAi animals, combined with the predicted function of *nhr-31* regulatory targets, provide several clues into how this nuclear receptor may impact the generation of a healthy EC (Figure 7). A critical component of EC development is the outgrowth of the excretory canals. During larval development, four excretory canals must grow out of the main cell body and migrate towards the posterior and anterior ends of the animal and then continue to grow as the animal increases in length. We observed that, during early larval development, the EC migrates along the length of the animal and is periodically punctuated with small oval shaped varicosities. By the time a worm reaches later larval stages, these varicosities are no longer present and adult EC canals are exquisitely uniform in diameter. We suggest that the growth varicosities that form during early larval development may be regions of high cellular growth activity, where robust protein, organelle, and membrane synthesis occur, these areas of growth then serve to supply material to the cytosol, as well as the basal and apical membranes of the EC, thus enabling the EC canal to elongate in a bidirectional manner. TEM images of the EC in L1 larvae, which show periodic varicosities with a more dense supply of membrane and organelles, support this hypothesis (Figure 5C and 5D). As the EC reaches its full-length, precise regulation of new cellular synthesis and cellular elongation reaches equilibrium such that regions of high EC cellular mass become evenly distributed and the EC adopts a fully mature and uniform shape.

Many of defects observed in *nhr-31* RNAi animals are consistent with an inability to properly regulate the coordination between EC cell outgrowth and new synthesis of cellular material. Thus, the NHR-31 nuclear receptor may play an important role in regulating the growth and elongation of the EC cell, and, in *nhr-31* RNAi animals, excess lipid synthesis and other factors involved in cellular growth proceed unchecked leading to the production of new EC cellular material, even as this cell is no longer growing lengthwise. In this scenario, actively growing regions of the excretory cell could not expand laterally in either direction; consequently, excess cellular material would accumulate in varicosities that continue to grow larger even after animals reach adulthood.

### A New Role for Nuclear Receptors in Control of vATPase Expression and Tube Morphogenesis

It is astonishing that NHR-31 controls such a small and specific set of target genes, and that nearly all of its targets comprise subunits or cofactors of the vATPase. While the fundamental conclusions of this study are not dependent upon the mechanism by which NHR-31 regulates gene transcription, NHR-31 is a transcription factor of the nuclear receptor type, and therefore it is tempting to propose that NHR-31 regulates the vATPases in response to a ligand signal by directly binding to the vATPase promoters. Consistent with this hypothesis, our binding site analyses of the vATPase promoters revealed a significant enrichment of nuclear receptor response elements in the form of ER6 or ER8 everted repeats with an AGTTCA consensus half site (Figure 6A and Table 4). The fact that this response element does not perfectly match the preferred response element architecture of the mammalian HNF4α is not surprising, as *C. elegans* contains dozens of HNF4-like receptors, and it is likely that NREs have evolved in nematodes in order to distinguish between NHR paralogs. We did, however, find strong enrichment of classical HNF4α binding sites (DR1 and DR2) in the promoters of the mouse vATPase genes, suggesting that coordinate regulation of the vATPase by HNF4 type receptors may be well conserved in mammals, even though the exact response element architecture may have changed.

The physiological functions and target genes of *nhr-31* have not been previously linked to an HNF4-type receptor, or any other nuclear receptor. NHR-31 shares a high degree of homology with mammalian HNF4 receptors, including nearly perfect conservation of key DNA binding elements and a strongly conserved ligand-binding domain (LBD). Interestingly, it has been proposed that the mammalian HNF4 receptors interact with free fatty acids and fatty acyl-CoA molecules [41],[42]. An ability of NHR-31 to bind to the acyl chain of a fatty acid or lipid molecule would provide a provocative explanation for how NHR-31 may be coordinating membrane synthesis and cellular elongation in the EC, which is likely to be occurring at sites distant from the nucleus. Because intensive membrane synthesis, transport, and fusion must take place in order to meet the needs of a growing excretory cell, such processes may release lipid based signals that activate or repress NHR-31 control of vacuolar ATPases and other genes associated with membrane biogenesis. Whether or not the functions of NHR-31 are conserved in mammals remains to be determined; however, the fact that both HNF4α and vacuolar ATPases are expressed at high levels in the proximal tubules of the mammalian kidney, combined with our demonstration that the mammalian vATPase genes contain a high density of HNF4α binding sites, implies that a functional role for HNF4 receptors in coordinate regulation of the vATPase in the renal system may indeed be a conserved process [16],[39].

## Materials and Methods

### *C. elegans* Strains and RNAi Constructs

The N2 Bristol strain of *C. elegans* was used for all experiments. Worms were maintained by standard techniques at 20–22°C. *nhr-31* RNAi constructs were created by introducing the full-length NHR-31 cDNA into the L4440 RNAi feeding vector (Andy Fire, Stanford University). RNAi constructs for *vha-5*, *vha-8*, and *vha-12* were obtained from the Ahringer RNAi library (University of Cambridge, Cambridge, UK). All RNAi constructs were transformed into the HT115 strain of *E. coli* and RNAi was introduced to N2 worms by RNAi feeding. RNAi expression was induced in the feeding bacteria by growing bacteria on NGM plates containing 3 mM IPTG and 100 µg/ml carbenicillin. Bacteria containing an empty L4440 RNAi vector were used for the RNAi control. Although NHR-31 is part of a large family of related nuclear receptors, these receptors have extensively diverged from one another during evolution, such that the closest paralog of NHR-31 shares only 55% homology in cDNA sequence; therefore, it is highly unlikely that there will be cross reactivity of the RNAi. Furthermore, *C. elegans* RNAi prediction programs do not indicate any cross reactivity (www.wormbase.org) [28]. Finally, the fact that *nhr-31(+/−)* heterozygotes displayed similar EC defects further supports the specificity of this RNAi construct (Figure S1).

### Construction of GFP Reporter Plasmid

An *nhr-31* promoter/gfp reporter construct (P*_nhr-31_*::gfp) was generated by fusing ∼3 kb of upstream regulatory sequence and 17 base pairs of the first *nhr-31* exon to the *gfp* gene, primers were created using the *nhr-31a.1* predicted isoform. Promoter DNA was amplified from genomic DNA using the following primers: NR-31UPGF (5′-TAA CTC GAG GAC GCA GGA AAG TCG GCA GTA GG-3′), as the 5′ upstream primer and NR-31-ExonI (5′-TCA CCC GGG TAC TCC CAA TCT TCG A-3′) as the 3′ downstream primer. Amplified DNA was inserted into the L3691 GFP reporter vector (from Andy Fire, Stanford University).

### Imaging and Measurement of the Excretory Cell by Fluorescence Microscopy

The P*_nhr-31_::gfp* reporter vector was introduced into N2 worms by microinjection at a concentration of 50 ng/µl, worms were selected by EC fluorescence and no co-injection marker was used. Worms harboring the P*_nhr-31_*::*gfp* transgene were examined by both standard fluorescence microscopy and confocal microscopy. Images were taken using AxioVision 4.6 software in multi-channel acquisition mode with an AxioCam MRU camera (Carl Zeiss Microimaging). For observation, larval or adult worms were mounted on glass slides with 2% agarose pads containing azide. Stack images of animals treated with *nhr-31*, *vha-5*, *vha-8 and vha-12* RNAi were taken in both the FITC channel (488 nm) and DIC channels.

To measure excretory cell diameter, control and *nhr-31* RNAi animals expressing P*_nhr-31_::gfp* (5–6 animals) were analyzed by taking images which captured 50–100 µm of the proximal, middle, or distal regions of the posterior excretory cell tube. Diameter measurements were taken every 4–5 µm within the imaged regions using Zeiss measurement software. Data were plotted using Origen 5.0 (OrigenLab, Northampton, MA) software, and data displayed in dot plots reflected values from each independent measurement, along with the mean, and standard deviation from the mean.

### High-Pressure Freezing Transmission Electron Microscopy

Day 2 adults or L1 larvae were placed into a 20% BSA/PBS buffer solution and prepared in a Leica-Impact-2 high-pressure freezer according to the following protocol: 1) 60 hours in 100% acetone and uranyl acetate at −90°C. 2) Temperature was ramped from −90°C to −25°C over the course of 32.5 hours. 3) Next, sample was incubated at −25°C for 13 hours. 4) Next, the temperature change was brought from −25°C to 27°C in a 13 hour temperature ramp. Serial sections were post-stained in uranyl acetate followed by lead citrate. Thin cross sections were taken from resin-embedded clusters of young adults or L1 larvae. Sections for *nhr-31* RNAi and control RNAi adult animals were obtained from 5 different animals, and sections for L1 larvae were also taken from 5 independent animals.

### Microarray and QRT–PCR

Synchronized L1 populations were prepared by hypochlorite bleaching of gravid N2 adults according to established protocols [6]. Synchronized L1 larvae were grown on control RNAi bacteria or *nhr-31* RNAi until animals reached the early L4 stage of development. Worms were then harvested in M9, washed five times and immediately frozen in liquid nitrogen. RNA was extracted using a TRIZOL based method as described [6]. RNA was then labeled with Cy3 or Cy5 and hybridized to Washington University manufactured *C. elegans* microarrays (http://genome.wustl.edu). Data were obtained from three independent biological replicates and analyzed using GenePix Pro 6.0 software (Molecular Devices, Sunnyvale, CA). Ratios were calculated using background corrected, and normalized data (global mean).

For QRT-PCR, RNA was extracted and cDNA was prepared using our previously published protocol [6] with the following exception: RNA was separated from genomic DNA with a Turbo DNA free prep kit from Ambion (Austin, TX). qPCR was performed using a BioRad iCycler (MyiQ Single Color, Bio-Rad Laboratories, Hercules, CA). The data were analyzed as previously described [6]. QRT-PCR primers amplified ∼100 base pair regions of NHR-31 target genes. Primers were designed using Primer3 software and calibrated by serial dilution of cDNA and genomic DNA. Primer sequences are available upon request.

### Salt Sensitivity Assays

Worms treated with control RNAi or *nhr-31* RNAi from the L1 to L4 stage of development were plated on high salt (500 mM NaCl) NGM-Lite plates seeded with *E. coli*. After various periods of high salt exposure, worms were scored for the ability to survive when rescued to a standard salt plate. Data for each time point was obtained from 250 animals. For rescue, worms were collected from the salt plates using M9 buffer+300 mM NaCl and transferred to standard NGM plates containing 50 mM NaCl. Worms were scored for survival after 12 hours of recovery [28].

### Nuclear Receptor Response Element Prediction

To identify putative nuclear receptor response elements (NREs), we use the online computer program NHR-scan (http://nhrscan.genereg.net), which was first presented in a study by Sandelin and Wasserman [34]. The promoters of *C. elegans* vATPase genes were defined as the sequence between the ATG translational start site of the *vha* gene of interest and the beginning or end of the next upstream gene in the *C. elegans* genome. For vATPase genes expressed in operons, the promoter was chosen using the ATG translational start site of the first gene in the operon. For mouse promoters, 2000 nucleotides of upstream sequence were extracted from each vATPase gene. This sequence included 1950 nucleotides upstream of the translational start site +50 nucleotides of coding sequence. In all cases, the isoform with the most 5′ translational start site was selected for promoter sequence extraction. To calculate and display the consensus half sites shown in Figure 6, all half site sequences were analyzed using the WebLogo online program (http://weblogo.berkeley.edu/logo.cgi) [43].

## Supporting Information

Figure S1 DIC image of the excretory cell of an nhr-31(+/−) mutant shows EC defects similar to those of nhr-31 RNAi animals.
(0.14 MB PDF)

Figure S2 Enlarged versions of the EM images shown in the text.
(10.93 MB PDF)

Table S1 The top upregulated (>3.5 fold) and downregulated (>2.5 fold) genes in nhr-31 RNAi animals.
(0.07 MB XLS)

Table S2 Of the top 30 downregulated genes, 15 of them encode subunits of the vacuolar ATPase.
(0.04 MB XLS)
