# Supplementary material for: The Caenorhabditis elegans HNF4α Homolog, NHR-31, Mediates Excretory Tube Growth and Function through Coordinate Regulation of the Vacuolar ATPase
Source: PLoS Genet. 2009 Jul 10;5(7):e1000553. doi: 10.1371/journal.pgen.1000553 (PMC2720251; doi:10.1371/journal.pgen.1000553)
Supplement: Figure S1 — DIC image of the excretory cell of an nhr-31(+/−) mutant shows EC defects similar to those of nhr-31 RNAi animals. (0.14 MB PDF) [file pgen.1000553.s001.pdf]

Figure S1

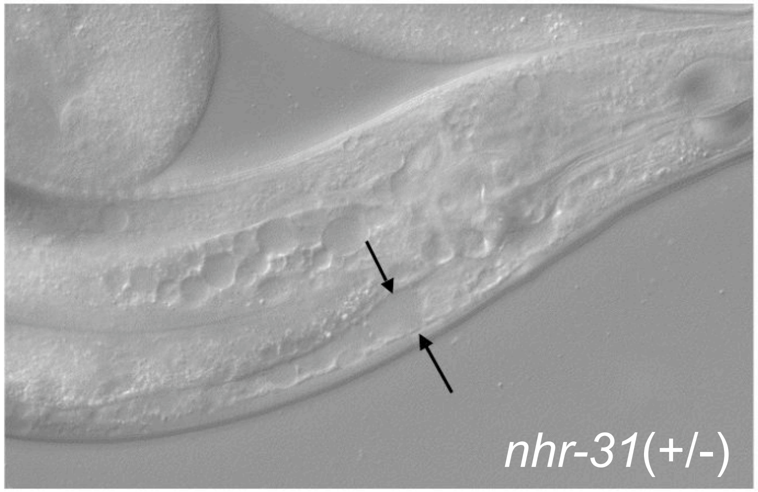

Arrows indicate excretory cell canal diameter is expanded, animal filled with vacuoles

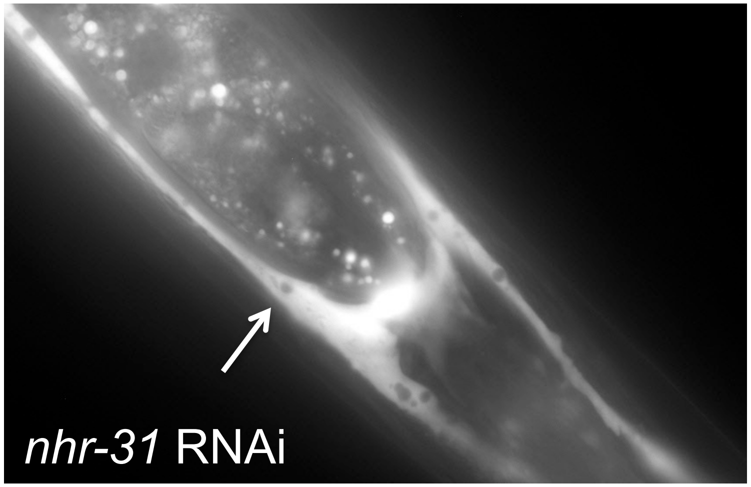

As shown in this young adult exposed to *nhr-31* RNAi, the EC is expanded with vacuoles in the proximal end of the canals
