# Supplementary material for: The Caenorhabditis elegans HNF4α Homolog, NHR-31, Mediates Excretory Tube Growth and Function through Coordinate Regulation of the Vacuolar ATPase
Source: PLoS Genet. 2009 Jul 10;5(7):e1000553. doi: 10.1371/journal.pgen.1000553 (PMC2720251; doi:10.1371/journal.pgen.1000553)

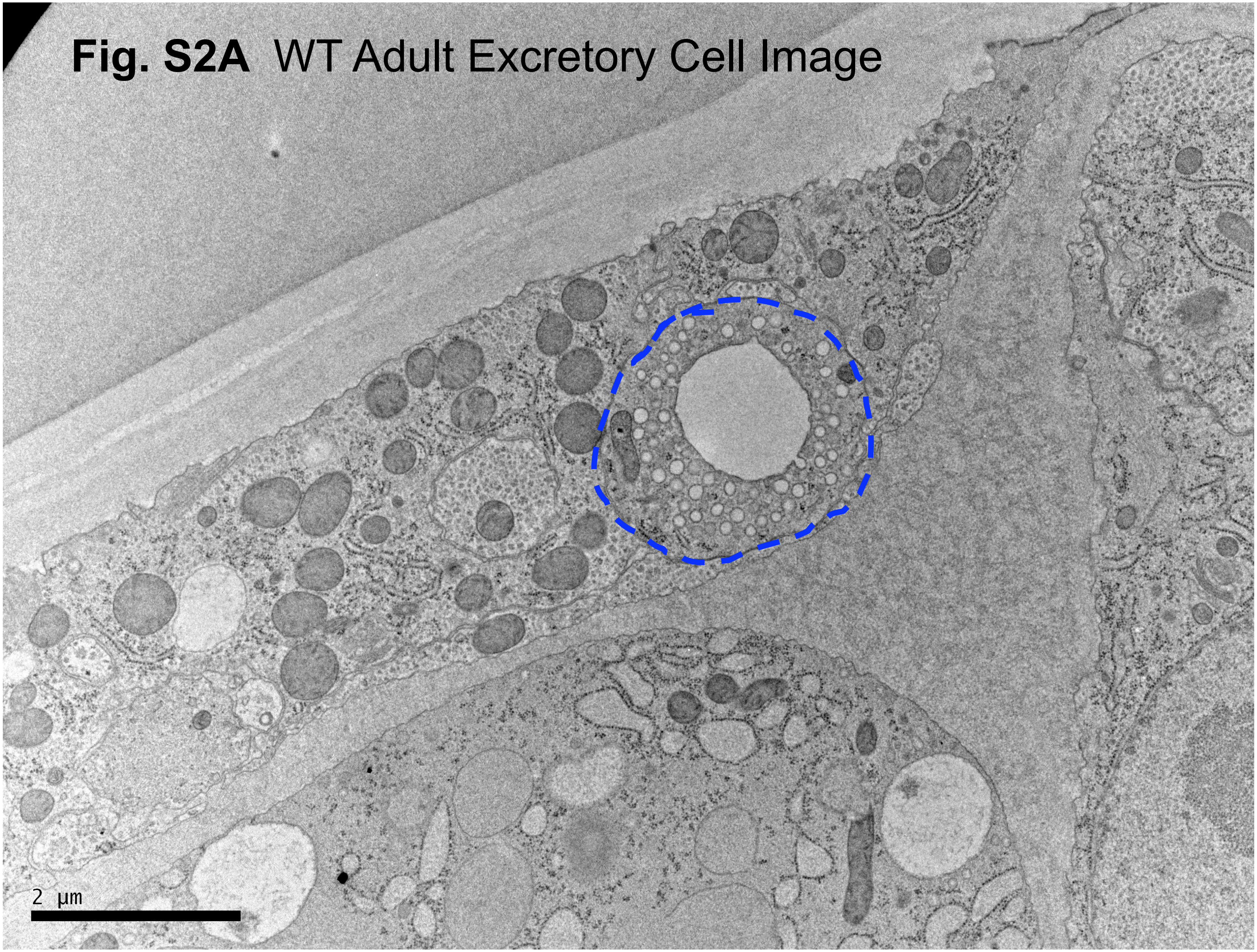
Fig. S2A WT Adult Excretory Cell Image
2 µm

Fig S2B *nhr-31* RNAi Adult Excretory Cell Sectioned through Varicosity

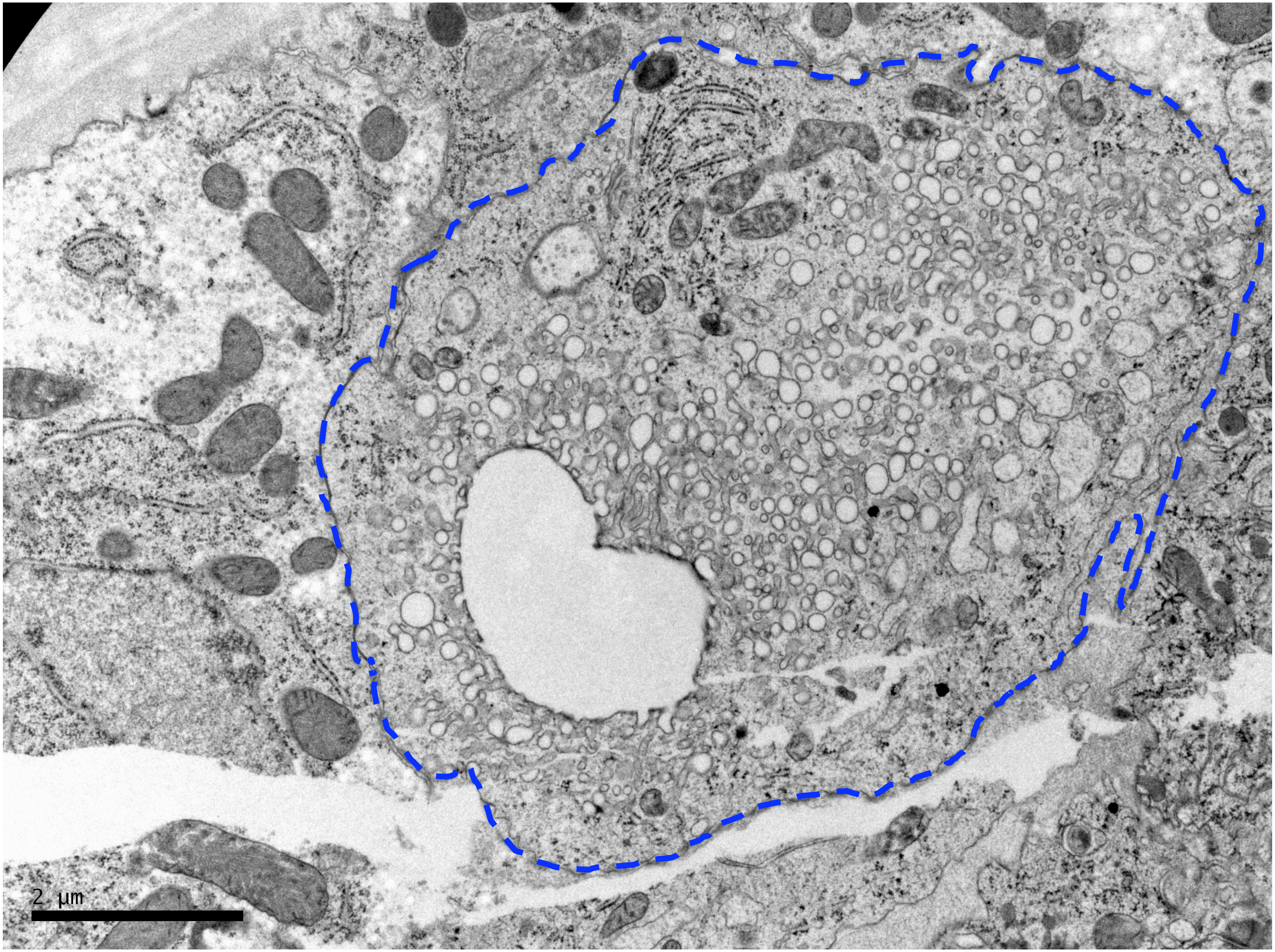

Fig S2C. WT L1 larvae Sectioned Through a Large Varicosity

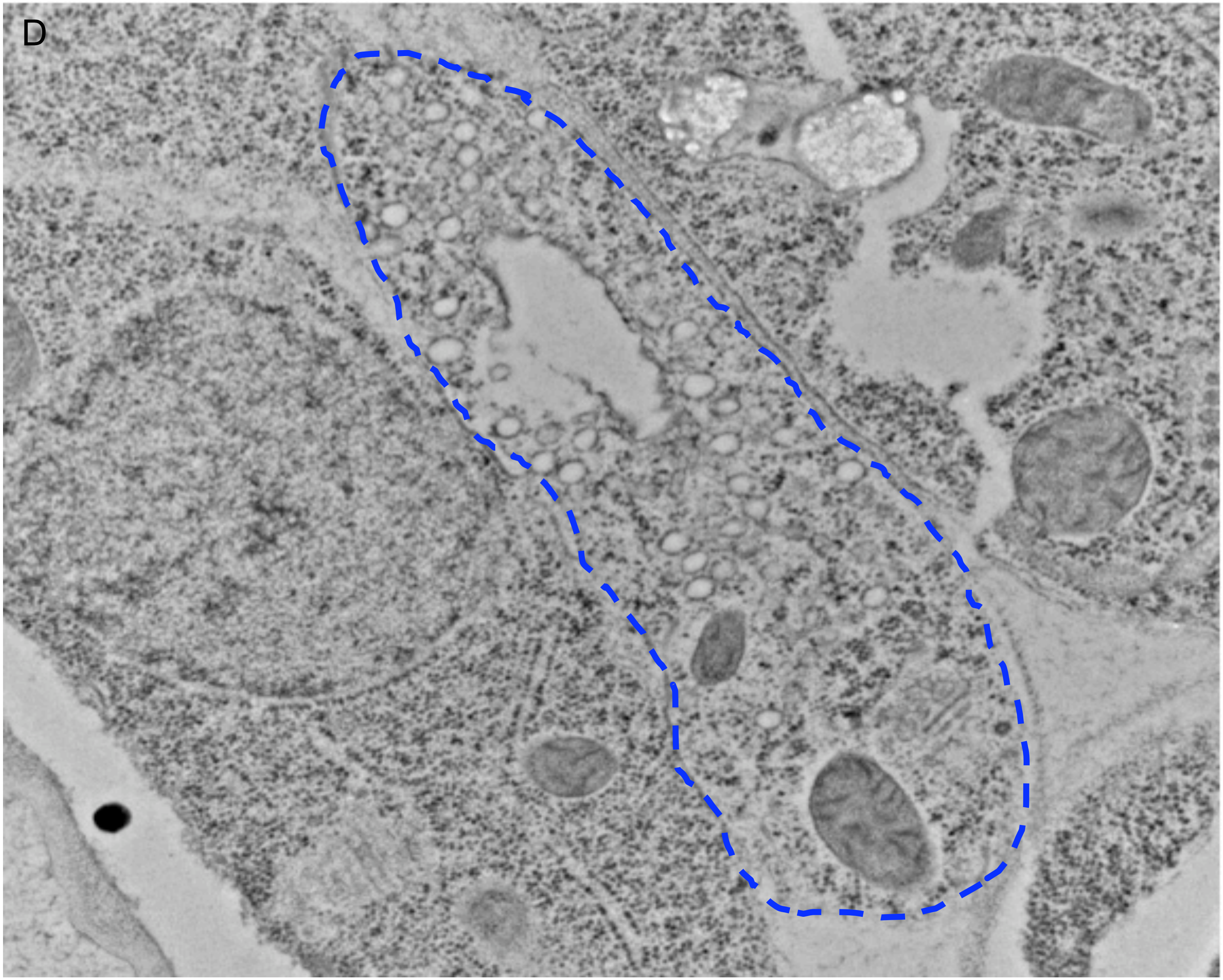

Fig. S2D. WT L1 Worm Sectioned Through a Narrow Region of the Excretory Cell

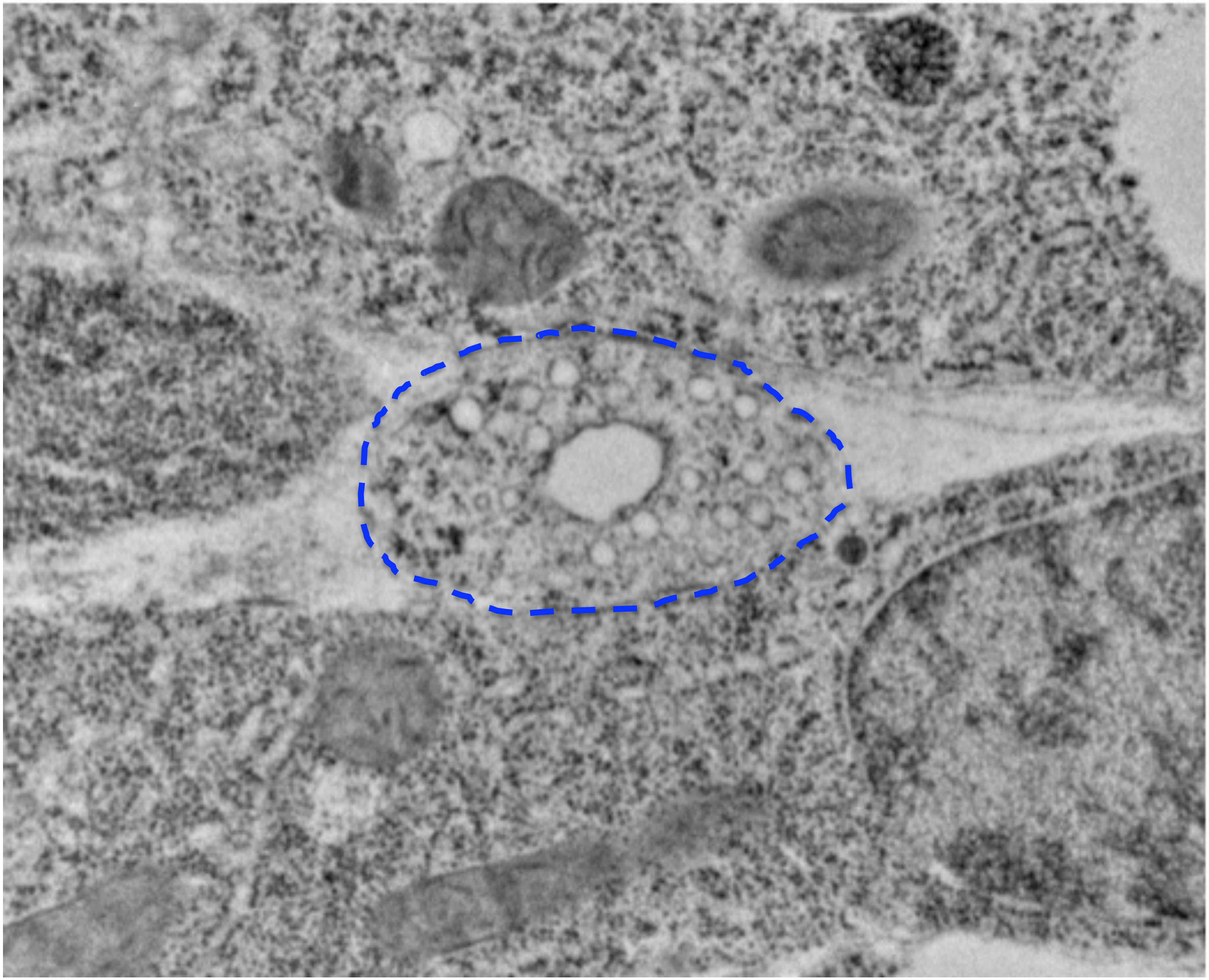

Supplement: Figure S2 — Enlarged versions of the EM images shown in the text. (10.93 MB PDF) [file pgen.1000553.s002.pdf]
